# Effectiveness of a soccer injury prevention program based on creatine supplementation and internal load monitoring: a randomized controlled pilot study

**DOI:** 10.1080/15502783.2026.2633251

**Published:** 2026-02-18

**Authors:** María Soler Hurtado, Moea Treguier, Ángel González-de-la-Flor, Diego Domínguez-Balmaseda, María García-Arrabé, Diego Miñambres Martin, Guillermo García-Pérez-de-Sevilla

**Affiliations:** a Universidad Europea de Madrid Faculty of Medicine, Health and Sports Department of Physiotherapy Campus de Villaviciosa, Madrid, Spain; b Universidad Europea de Madrid Faculty of Medicine, Health and Sports Department of Real Madrid Graduate School, Campus de Villaviciosa, Madrid, Spain

**Keywords:** Creatine supplementation, injury prevention, soccer, male, muscle strength

## Abstract

**Background:**

Musculoskeletal injuries are frequent in soccer, occurring more often in matches than in training sessions, and mainly affecting the lower limbs. While creatine supplementation is commonly used to enhance performance, evidence regarding its role in injury prevention among soccer players remains limited. This pilot study investigated whether creatine supplementation could reduce the incidence of musculoskeletal injuries in amateur soccer players.

**Methods:**

A 14-week randomized controlled pilot trial was conducted with 23 amateur soccer players randomly assigned to the creatine group (*n* = 12; 3 g/day) or the placebo group (*n* = 11; maltodextrin). Primary outcomes were injury incidence and training availability. Secondary outcomes included isometric strength, countermovement jump (CMJ) performance, perceived pain, well-being, and internal load. Assessments were performed at baseline (January 2025) and post-intervention (April 2025).

**Results:**

Twenty-four players were randomized (12 creatine, 11placebo). Injury incidence was lower with creatine (8.3%) than placebo (36.4%) (RR = 0.23, 95% CI 0.03–1.75; *p* = 0.155). Missed training sessions were fewer with creatine (0.25 ± 0.87 vs 0.82 ± 1.40; *p* = 0.135). Significant time × group effects favored creatine for knee extension, knee flexion, hip extension, right hip flexion, and CMJ height (all *p* < 0.01).

**Conclusion:**

Creatine supplementation improved strength and jump performance and showed a trend toward lower injury incidence, supporting its potential as an adjunct in soccer injury-prevention programs.

## 1 Introduction

Musculoskeletal injuries represent a major health concern in soccer, with substantial implications for athletes’ biopsychosocial performance and team dynamics [1,2]. Epidemiological evidence indicates a markedly higher injury incidence during competition (8.7–65.9 per 1000 hours) compared with training (1.37–5.8 per 1000 hours), with the majority affecting the lower limbs [3].

Injury risk is multifactorial and influenced by intrinsic factors—such as age, prior injury history, and fatigue—as well as extrinsic factors including playing surface and seasonal demands [4–6]. Injury prevention therefore requires a coordinated, multidisciplinary approach. Medical doctors, physiotherapists, strength and conditioning coaches, nutritionists, and sport psychologists each play a pivotal role in safeguarding athlete health and optimising performance [7,8]. Beyond performance, this support team carries an ethical responsibility to ensure safe return-to-play, even when competitive pressures may challenge medical oversight [9,10].

Training load management is a critical determinant of injury risk. Both excessive and insufficient loads can predispose athletes to injury. In soccer, repeated high-intensity efforts without adequate recovery—often requiring >72 hours for restoration of muscle glycogen and normalisation of creatine kinase—create conditions of overload [11]. Match congestion, high physical demands, and the movement patterns intrinsic to the sport further compound this risk. Recovery strategies such as cold-water immersion, massage, and optimised sleep have been widely advocated to mitigate fatigue and reduce injury risk [12–15].

Fatigue, defined as the inability to maintain a given level of force or intensity [16], is a key mediator of injury susceptibility. At the physiological level, fatigue arises when adenosine triphosphate (ATP) consumption exceeds resynthesis capacity, leading to depletion of phosphocreatine (PCr) stores and impaired energy metabolism [17].

Creatine supplementation has emerged as a potential strategy to enhance performance and accelerate recovery [18,19]. Creatine, a compound synthesised from amino acids, increases intramuscular phosphocreatine (PCr) availability, thereby facilitating rapid adenosine triphosphate (ATP) resynthesis during repeated high-intensity efforts [20]. From a mechanistic perspective, enhanced PCr availability may attenuate fatigue-related declines in force production, support neuromuscular function, and improve tolerance to training loads. In addition, creatine has been proposed to influence cellular hydration, thermoregulation, and post-exercise recovery processes, which may indirectly contribute to reduced injury susceptibility and improved tissue resilience [21,22]. Nevertheless, evidence supporting a direct preventive effect of creatine on musculoskeletal injury incidence in soccer remains limited and inconclusive.

The primary aim of this randomised controlled pilot study was to investigate whether creatine supplementation can reduce the incidence of musculoskeletal injuries and improve training availability in amateur soccer players, thereby contributing to improved athlete health and continuity of training exposure across the competitive season. Given the central role of fatigue-related mechanisms and phosphocreatine availability in injury susceptibility, this study also sought to explore creatine supplementation as a practical, low-cost nutritional strategy within a multidisciplinary injury risk management framework. Secondary aims were to examine the effects of creatine on internal training load, lower-limb strength, and vertical jump performance. We hypothesised that creatine supplementation would result in lower injury incidence and pain perception, as well as greater training availability compared with placebo over a 14-week intervention period.

## 2 Methods

### 2.1 Study design and ethics

A randomised, controlled, parallel-group pilot trial was conducted over a 14-week period (January–April 2025). The study design adhered to the CONSORT guidelines for pilot and feasibility studies [23].

The protocol was approved (30/12/2024) by the Ethics Committee of Hospital Clínico San Carlos (Ref. 24/839-E). Written informed consent was obtained from all participants prior to enrolment, in accordance with the Declaration of Helsinki and current data protection regulations. This study was registered with the Australian New Zealand Clinical Trials Registry (ACTRN 12624001437550) on December 12th, 2024 (https://anzctr.org.au/).

### 2.2 Participants

Participants were male soccer players competing in the league (amateur level) in Madrid, Spain. Inclusion criteria were age 18–35 years, absence of musculoskeletal injury in the previous four months that would prevent training or match participation, and no history of renal disease. A convenience sample was recruited due to the pilot nature of the study.

### 2.3 Randomisation and blinding

A total of 24 players were recruited and randomly assigned in a 1:1 ratio to either the intervention group (creatine) or the placebo group (maltodextrin). Randomisation was performed using the online tool *AppSorteos*. A single-blind design was applied: participants were blinded to group allocation, while the supervising staff were aware of assignments.

### 2.4 Protocol

Participants in the creatine group (CG) received 3 g/day of creatine monohydrate (Creapure®) five days per week. Supplementation was administered immediately after training sessions or with a meal on rest days, under the supervision of a certified nutritionist. Participants in the placebo group (PG) received an equivalent dose of maltodextrin, a non-ergogenic substance with similar appearance and taste to creatine.

This creatine dose was selected based on recommendations from previous studies in athletes, and specifically in soccer players, which indicate that when supplementation is intended to be maintained over the medium term, a daily dose of 3–5 g of creatine is appropriate, sufficient, and beneficial for improving sports performance [24,25]. Several studies have shown no significant differences between doses close to 3 g/day and 5 g/day in terms of physical performance; therefore, the lower dose (3 g/day) was chosen to maximise participant safety [26,27].

The intervention lasted 14 weeks, during which both supplements were provided by Zumub® (Lisbon, Portugal). All evaluations were conducted at two time points, baseline (T1) and post-intervention (T2), to determine the effects of the intervention on performance, injury incidence, and well-being (Table 1). At the beginning of the study, baseline characteristics were recorded, including age, body mass, height, and body mass index (BMI). In addition, information related to injury incidence and player availability was collected, where athletes reported any absence from training or competition due to lower-limb musculoskeletal injuries, and epidemiological data such as location, type, recovery time, and injury mechanism were documented. Adherence to supplementation was monitored weekly through self-reported intake logs, while voluntary withdrawal was permitted at any time without penalty.

**Table 1.** Outcome measures and assessments.

Outcome	Instrument/Method	Description/Measurement details
Anthropometric variables	Standard anthropometry	Age, body mass, height, and BMI measured at baseline (T1).
Injury incidence	Self-reported injury log	Record of absences from training/competition due to lower-limb musculoskeletal injuries; includes location, type, recovery time, and mechanism.
Supplement adherence	Self-reported log	Weekly record of supplement intake; voluntary withdrawal allowed.
Maximal isometric strength	*ActivForce 2* dynamometer (ActivBody Inc., USA)	Bilateral assessment of knee and hip flexors and extensors; two 6-s maximal contractions per movement.
Lower-limb power	*My Jump 2* app (Apple Inc., USA)	Countermovement Jump (CMJ) test; video-based analysis of take-off and landing frames to estimate jump height.
Hip mobility (ROM)	*ActivForce 2* digital inclinometer	Active range of motion: hip flexion (supine, knee extended) and hip extension (prone).
Internal training load	Foster index	Calculated as session RPE (Borg CR-10 scale) × session duration (minutes).
Pain	Visual Analogue Scale (VAS, 0–10)	Self-reported pain intensity; 0 = no pain, 10 = maximal imaginable pain.
Well-being	Wellness Questionnaire (0–7 scale)	Ratings of stress, fatigue, sleep quality, and muscle soreness; higher scores indicate better condition.

#### 2.4.1 Maximal isometric strength

Maximal isometric strength was evaluated bilaterally using the ActivForce 2 digital dynamometer (ActivBody Inc., San Diego, CA, USA) for the assessment of knee and hip flexors and extensors. Each participant performed two 6-seconds maximal voluntary contractions per movement, separated by 6-second rest intervals, and the highest value obtained was used for analysis [28,29]. For the evaluation of knee flexors, participants were positioned prone with the knee flexed over a 12-centimetre foam roller, and the dynamometer was placed on the posterior aspect of the distal leg just above the Achilles tendon. For knee extensors, participants were seated with the hip and knee at 90 degrees and no back support, and the device was positioned on the anterior aspect of the distal tibia just above the ankle joint. Hip flexor strength was measured in a standing position with the tested limb flexed to 90 degrees at the hip and the dynamometer placed on the anterior aspect of the thigh proximal to the knee, while hip extensor strength was assessed with the participant standing and holding onto a treatment table for balance, the tested leg extended at the hip with the knee straight, and the dynamometer positioned on the posterior aspect of the thigh.

#### 2.4.2 Lower-limb power

Lower-limb power was assessed through the Countermovement Jump (CMJ) test using the My Jump 2 mobile application (Apple Inc., Cupertino, CA, USA). Participants performed three maximal jumps with their hands on their hips to minimise arm contribution, and the best jump height was retained for analysis. All jumps were recorded on video, and the frames corresponding to take-off and landing were analysed using a fixed floor reference to determine flight time and calculate jump height [30].

Before the assessment of maximal isometric strength and lower-limb power, participants completed a standardised 10-minute specific warm-up that included mobility exercises, low-volume strength exercises targeting the core and lower limbs, plyometric activities, and familiarisation with the CMJ technique.

#### 2.4.3 Hip mobility

Hip mobility was evaluated by measuring the active range of motion using the ActivForce 2 digital inclinometer. Hip extension was assessed in the prone position with the tested leg extended, while hip flexion was measured in the supine position with the knee fully extended.

#### 2.4.4 Internal training load

Internal training load was determined using the Foster index, calculated as the product of the session rating of perceived exertion (RPE, Borg CR-10 scale) and the total session duration in minutes, providing an estimate of the overall internal load imposed during training sessions [31].

#### 2.4.5 Pain and well-being

Pain perception was assessed using a Visual Analogue Scale (VAS) ranging from zero to ten, where zero indicated no pain and ten represented the maximum imaginable pain [32], while overall well-being was evaluated through a Wellness Questionnaire that rated stress, fatigue, sleep quality, and muscle soreness on a scale from zero to seven, with higher scores reflecting a better perceived condition [33].

### 2.5 Statistical analysis

Normality was examined with the Shapiro–Wilk test. Descriptive data are presented as mean ± SD for continuous variables and as frequencies (%) for categorical variables. Group differences in injury occurrence (yes/no) were assessed with risk ratios (RR), odds ratios (OR), and risk differences (RD) with 95% confidence intervals (CI), complemented by Fisher’s exact test and χ². Missed training sessions and matches were compared between groups using independent-samples t-test or Mann-Whitney U test depending on distributional assumptions.

For isometric strength and CMJ, a two-way mixed model (time × group) was conducted. Partial eta-squared (ηp²) was reported as effect size, interpreted as small (0.01), medium (0.06), or large (≥0.14). Secondary outcomes (internal load, pain, well-being) were analysed with independent-samples tests. Analyses were performed wtih SPSS v29 and significance level was set at *p* 0.05.

## 3 Results

Twenty-four players were randomised; one participant in the placebo group withdrew, leaving 12 in the creatine and 11 in the placebo group. At baseline, age was 27.73 ± 2.87 years in the placebo group and 27.50 ± 1.68 years in the creatine group (*p* = 0.817), while height was 180.82 ± 4.56 cm and 177.75 ± 5.64 cm, respectively (*p* = 0.169).Groups were comparable at baseline for age, anthropometrics, and most strength outcomes, although hip flexion strength was higher in placebo (*p* = 0.022–0.027). At baseline, differences were observed for left and right hip flexion strength (*p* = 0.022 and *p* = 0.027, respectively), whereas no significant between-group differences were found for the remaining variables (all *p* > 0.05).

Injury incidence was lower in creatine (1/12; 8.3%) than placebo (4/11; 36.4%), corresponding to RR = 0.23 (95% CI 0.03–1.75), OR = 0.16 (95% CI 0.01–1.73), and RD = –28.0% (95% CI –60.5 to + 4.4). Fisher’s exact *p* = 0.155. Missed training availability was 0.25 ± 0.87 sessions in creatine vs 0.82 ± 1.40 in placebo (*p* = 0.135).

Significant time × group interactions in favour of creatine were found for bilateral knee extension (left *p* = 0.001; right *p* = 0.002), left knee flexion (*p* = 0.005), bilateral hip extension (*p* = 0.002), and right hip flexion (*p* = 0.003). CMJ height also increased significantly in the creatine group (*p* = 0.005). No between-group differences were found for pain (*p* = 0.583), well-being (*p* = 0.269), or internal load (*p* = 0.442). Weekly questionnaire adherence was 94.3% (Table 2).

**Table 2.** Anthropometric and performance outcomes (mean ± SD) at pre- and post-intervention.

Variable	Placebo Group PRE	Placebo Group POST	Intervention Group PRE	Intervention Group POST	*p*-value (time)	*p*-value (time × group)	ηp² (time × group)
Body mass (kg)	81.73 ± 9.56	80.91 ± 9.16	74.92 ± 7.39	76.42 ± 7.73	0.230	<0.001	0.457
BMI (kg/m²)	24.91 ± 1.80	24.67 ± 1.69	23.69 ± 1.80	24.16 ± 1.76	0.201	<0.001	0.436
Isometric knee extension L (kg)	26.11 ± 8.99	26.03 ± 9.46	26.32 ± 8.79	27.76 ± 8.38	0.003	0.001	0.407
Isometric knee flexion L (kg)	34.75 ± 9.85	34.64 ± 9.82	34.94 ± 4.12	35.96 ± 4.80	0.020	0.005	0.317
Isometric hip extension L (kg)	17.94 ± 6.30	17.60 ± 6.29	16.30 ± 5.89	17.06 ± 5.60	0.187	0.002	0.372
Isometric hip flexion L (kg)	22.49 ± 9.97	22.37 ± 10.10	15.19 ± 2.39	15.77 ± 2.60	0.184	0.054	0.165
Isometric knee extension R (kg)	25.58 ± 8.70	25.88 ± 8.97	25.31 ± 7.25	28.26 ± 8.30	<0.001	0.002	0.360
Isometric knee flexion R (kg)	35.59 ± 8.50	35.55 ± 8.00	36.30 ± 5.45	36.42 ± 4.68	0.903	0.801	0.003
Isometric hip extension R (kg)	18.27 ± 5.54	17.97 ± 5.50	15.80 ± 5.73	16.77 ± 5.81	0.075	0.002	0.375
Isometric hip flexion R (kg)	20.74 ± 7.35	20.51 ± 7.36	15.31 ± 2.88	16.01 ± 2.67	0.101	0.003	0.352
CMJ height (cm)	33.71 ± 4.82	33.38 ± 4.83	31.34 ± 3.02	31.98 ± 2.80	0.323	0.005	0.317
Hip extension ROM (°)	10.70 ± 2.26	10.77 ± 2.28	10.40 ± 2.20	10.59 ± 2.12	0.067	0.394	0.035
Hip flexion ROM (°)	60.87 ± 10.87	61.27 ± 10.57	62.35 ± 9.94	62.92 ± 10.19	0.014	0.648	0.010

Abbreviations: BMI, body mass index; CMJ, countermovement jump; ROM, range of motion; L, left; R, right; PRE, pre-intervention; POST, post-intervention; ηp², partial eta squared.

### 3.1 Post-hoc analysis

The creatine group demonstrated larger gains in knee extension strength (left ΔMD = + 1.7 kg, 95% CI 0.7–2.6; right ΔMD = + 2.4 kg, 95% CI 1.3–3.5), left knee flexion strength (ΔMD = + 1.0 kg, 95% CI 0.2–1.8), left and right hip extension strength (ΔMD = + 1.2 to + 1.5 kg, 95% CI 0.5–2.4), and right hip flexion strength (ΔMD ≈ + 0.7 kg, 95% CI 0.1–1.3), as well as CMJ height (ΔMD = + 0.6 cm, 95% CI 0.2–1.1). In contrast, no significant differences were observed for hip flexion left, right knee flexion, or hip range of motion.

## 4 Discussion

This pilot randomised trial provides preliminary evidence that creatine supplementation (3 g/day) during a competitive soccer season improves lower-limb isometric strength and CMJ performance and may contribute to a reduction in injury incidence and training availability loss. Although the between-group differences in injuries did not show statistical significance, the magnitude of the observed effect (RR 0.23; OR 0.16) suggests a potentially relevant clinical benefit.

Regarding injury prevention, our findings are complementary to those of Varillas-Delgado et al. [34], who examined professional football players and demonstrated that the response to creatine supplementation in terms of muscle mass gain and injury prevention was strongly influenced by genetic polymorphisms. In that cohort, athletes with a favourable total genotype score (TGS) showed significantly greater increases in muscle mass and a lower risk of non-contact injuries, whereas those with lower TGS values were more prone to injuries despite supplementation. While our pilot trial in amateur players did not assess genetic factors, the observed improvements in lower-limb strength, CMJ performance, and the directional reduction in injury incidence align with the mechanistic basis reported by Varillas-Delgado et al. [34] suggesting that creatine supplementation enhances neuromuscular performance and may contribute to injury prevention.

It is important to consider that elite players typically present higher baseline levels of neuromuscular performance and training-induced muscle hypertrophy compared with amateur or less-trained athletes. Recent evidence indicates that training status may influence the nature of fat-free mass gains associated with creatine supplementation. The body mass gains observed in untrained individuals may be partially attributable to factors other than muscle hypertrophy, such as fluid retention. These findings highlight that training history may modulate the qualitative characteristics of creatine-induced adaptations and should be considered when extrapolating results between amateur and elite soccer players [35].

The present findings are supported by recent metanalyses showing that creatine supplementation, particularly when combined with structured training, significantly enhances squat performance with a weighted mean difference of 5.64 kg and minimal heterogeneity (I² = 4.1%) [36]. Subgroup analyses revealed that these effects were most pronounced in younger, male, and previously trained individuals, as well as with higher daily maintenance doses and greater training frequency, underscoring the dose–response and context-specific nature of creatine’s ergogenic benefits. Our data, demonstrating significant gains in lower-limb isometric strength and jump performance, are consistent with these findings and reinforce the notion that creatine augments neuromuscular adaptations to training. Importantly, the meta-analysis also suggested that short-term interventions (<8 weeks) produced the greatest strength gains, which aligns with the early-phase improvements observed in our pilot trial and highlights the potential for creatine to enhance initial neuromuscular adaptations in competitive settings.

The ergogenic effect of creatine observed in this study is consistent with extensive literature demonstrating increased intramuscular phosphocreatine content, enhanced ATP resynthesis, and improved performance in high-intensity, short-duration efforts [26,37]. These adaptations are particularly relevant in soccer, where repeated sprints, accelerations, and eccentric actions place athletes at high risk of fatigue-related injuries. Several trials in team-sport athletes have shown that creatine supplementation improves strength, sprint ability, and jump performance [18,38].

Finally, the null findings in internal load, pain, and well-being scores may reflect the limited sensitivity of these measures in short competitive windows or the modest supplementation dose used. Importantly, no adverse effects were reported, supporting the excellent safety profile of low-dose creatine, as documented across several clinical and athletic populations [38].

### 4.1 Limitations

Several limitations should be acknowledged. The small sample size and single-team design limit statistical power and generalisability. The trial was single-blind, which may introduce performance-related bias despite supervised intake. A baseline imbalance in hip flexion strength favoured the placebo group and may have influenced post-intervention contrasts. Injury outcomes were reported as counts without adjustment for individual exposure (training and match hours), which precludes standardised incidence rates and may distort comparisons, as consensus recommendations for football injury surveillance emphasise the need to quantify exposure to interpret injury risk appropriately [39].

Several potentially relevant confounders were not systematically monitored. Dietary intake beyond supplementation (total energy/protein, carbohydrate periodisation), hydration and water intake, and the use of other ergogenic aids were not controlled and may influence both performance and recovery. In addition, physical activity performed outside team sessions, individual recovery strategies, and sleep quantity/quality were not recorded; these factors are known to affect neuromuscular performance and may also influence injury risk, particularly when sleep is insufficient [40]. Furthermore, prior injury history was not fully characterised, despite its recognised influence on subsequent injury risk and player availability. External load and training/match demands were not quantified in detail (e.g. acute–chronic workload changes, high-speed running exposure), which is relevant given evidence linking training load patterns to injury risk [41]. Finally, contextual factors such as field type and playing conditions across the season (e.g. natural grass vs artificial turf, weather, surface hardness) were not assessed. Our findings should be considered with caution when interpreting the observed effects on performance and injury outcomes. Therefore, future studies should incorporate systematic monitoring of exposure, training load, diet/hydration, sleep, and previous injury history to reduce residual confounding and strengthen causal inference.

## 5 Conclusions

Creatine supplementation was associated with improvements in selected measures of neuromuscular performance in amateur soccer players.

A lower incidence of injuries was observed in the supplemented group; however, due to the lack of full control over potentially confounding variables and the inherently multifactorial aetiology of sports injuries, causality cannot be established.

Injury risk in team sports is influenced by complex interactions among training load, recovery status, match exposure, individual characteristics, and contextual factors. These variables were not fully controlled and may have influenced the observed outcomes.

Therefore, the present findings should be interpreted with caution. Further prospective, adequately controlled studies are required to clarify the magnitude and mechanisms of the association between creatine supplementation and injury risk.

Within an integrated, evidence-informed performance and injury-prevention framework, creatine supplementation may be considered as a complementary strategy rather than a standalone intervention.

## Data Availability

De-identified individual participant data (including data dictionary), statistical code, and any other study materials are available from the corresponding author upon reasonable request.
